# Enhanced Video Anomaly Detection Through Dual Triplet Contrastive Loss for Hard Sample Discrimination

**DOI:** 10.3390/e27070655

**Published:** 2025-06-20

**Authors:** Chunxiang Niu, Siyu Meng, Rong Wang

**Affiliations:** College of Information and Cyber Security, People’s Public Security University of China, Beijing 100038, China; 2023211460@stu.ppsuc.edu.cn (C.N.); 2023211536@stu.ppsuc.edu.cn (S.M.)

**Keywords:** abnormal behavior detection, multiple instance learning, hard instance, contrastive loss function, multi-scale feature

## Abstract

Learning discriminative features between abnormal and normal instances is crucial for video anomaly detection within the multiple instance learning framework. Existing methods primarily focus on instances with the highest anomaly scores, neglecting the identification and differentiation of hard samples, leading to misjudgments and high false alarm rates. To address these challenges, we propose a dual triplet contrastive loss strategy. This approach employs dual memory units to extract four key feature categories: hard samples, negative samples, positive samples, and anchor samples. Contrastive loss is utilized to constrain the distance between hard samples and other samples, enabling accurate identification of hard samples and enhancing the discriminative ability of hard samples and abnormal features. Additionally, a multi-scale feature perception module is designed to capture feature information at different levels, while an adaptive global–local feature fusion module constructs complementary feature enhancement through feature fusion. Experimental results demonstrate the effectiveness of our method, achieving AUC scores of 87.16% on the UCF-Crime dataset and AP scores of 83.47% on the XD-Violence dataset.

## 1. Introduction

Video anomaly detection (VAD) [1] aims to detect and localize abnormal events in untrimmed videos, such as riots, robberies, and fights, which deviate from conventional patterns or established norms. As an extension of deep learning technology in the field of video surveillance, VAD has been widely applied to venue surveillance video analysis, crime prevention, and public safety alerts.

Currently, weakly supervised learning has emerged as the mainstream framework for VAD tasks [2], requiring only video-level labels for the training set. This approach effectively addresses the labor-intensive and time-consuming challenges associated with frame-level annotation of large-scale video datasets. Notably, the multiple instance learning (MIL) method introduced by Sultani et al. [3] represents a commonly adopted strategy in weakly supervised paradigms. Under this framework, each video sample is treated as a bag, where videos labeled as anomalous are treated as positive bags and normal videos as negative bags. Each video is segmented into non-overlapping fragments, referred to as instances. Subsequently, each instance is assigned an abnormality score, and the model is trained using the Top-K ranking loss to differentiate between normal and abnormal instances. Methods [4,5,6,7] further optimize loss strategies by clustering similar features and separating dissimilar features to enhance the discriminative power of feature representations. Chen et al. [8] address the cross-scenario feature magnitude misalignment issue by designing a joint magnitude contrastive loss function to construct dynamic spatiotemporal representations. However, existing weakly supervised approaches focus on learning discriminative features between abnormal and normal instances by emphasizing only those with the highest anomaly scores, while neglecting the mining and differentiation of hard samples, leading to misjudgments and a high false alarm rate. Hard samples [9] refer to normal instances that exhibit visual-semantic similarities to abnormal patterns due to video content complexity and semantic ambiguity [10,11], such as legitimate collisions or intense movements in sports events. To this end, constructing a mining method for hard samples and distinguishing hard samples from abnormal features can effectively address the false alarm issues caused by hard samples.

To address the aforementioned challenges of hard sample mining and misclassification, we propose the dual triplet contrastive loss strategy. By feeding the features of normal and abnormal videos into dual memory units, respectively, four critical features are extracted: negative samples, anchor samples, positive samples, and hard samples. The contrastive loss constrains the feature distances between the anchor sample and the positive sample, as well as between the anchor sample and the hard sample, to be minimized, while maximizing the feature distances between the anchor sample and the negative sample. This mechanism achieves accurate identification of hard sample features and distinction from abnormal features, thereby enhancing the discriminative capability between normal and abnormal features. Meanwhile, given the significant variations in object scales and the wide coverage of scenes in video scenarios, traditional single-scale feature extraction methods prove inadequate for effective adaptation, resulting in insufficient robustness of the model to scale changes and limited discriminative performance. To this end, the multi-scale feature perception (MFP) module is designed. This module employs a parallel multi-branch convolutional structure to extract features with different receptive fields and utilizes learnable channel attention to fuse feature information from different branches, capturing multi-level semantic information more comprehensively. Furthermore, the spatiotemporal information in video data exhibits inherent complexity where both global dynamic patterns and local fine-grained variations are critical for robust feature recognition. To effectively capture such complementary information, the adaptive global–local feature fusion (AGLF) module is constructed. This module employs the multi-head self-attention branch to capture global dynamic features among long-distance video segments and exploits the adaptive graph convolution branch to mine interrelationships of video frames within local neighborhoods, ultimately achieving complementary feature enhancement through fusion.

In summary, the contributions of this paper are as follows:

- A dual-triplet contrastive loss strategy is proposed to enhance the distinction between hard samples and abnormal features. This strategy extracts four types of sample features through dual memory units to enhance the distinction between hard samples and abnormal features.
- A multi-scale feature perception module is designed to capture feature information at different levels through a parallel multi-branch convolutional structure, and a learnable channel attention mechanism is utilized to fuse these features, thereby enhancing the discriminative ability of the model.
- An adaptive global–local feature fusion module is constructed, which employs two types of branch structures to separately capture global dynamic patterns and local fine-grained features, and a feature fusion strategy is implemented to achieve complementary feature enhancement.

## 2. Related Work

### 2.1. Multi-Scale Feature Extraction

Multi-scale features enhance model robustness and discriminative capabilities. Gong et al. [12] proposed a multi-scale continuity-aware refinement network to detect and maintain temporal continuity in videos at different scales. Ristea et al. [13] introduced a self-supervised predictive dilated convolutional attention block to expand receptive fields and capture multi-scale spatiotemporal features across video frames. Furthermore, Gong et al. [14] achieved continuous feature integration from shallow details to deep abstractions by constructing a spatiotemporal feature pyramid network with dilated convolution layers, layer by layer. Tian et al. [15] combined dilated convolutions with self-attention mechanisms, training a feature magnitude learning function to achieve hierarchical feature transformations from pixel-level to high-level semantic representations. Purwanto et al. [16] developed a relation-aware feature extractor to capture multi-scale convolutional features, coupled with conditional random fields to model multi-level feature dependencies. Additionally, Rahimpour et al. [17] designed an attention-guided spatiotemporal feature extractor that collaborates with an LSTM autoencoder to characterize multi-level motion patterns.

### 2.2. Local–Global Relationship Modeling

Temporal modeling methods are pivotal for capturing contextual information in videos and are broadly categorized into local relation modeling and global relation modeling [2]. Pu et al. [18] developed a local-aware attention network with spatiotemporal dynamic masks to enhance the representation of micro-motion features in adjacent segments. Naji et al. [19] established mappings between local spatiotemporal features and normal patterns through constrained pretraining. For global dependency modeling, Li et al. [20] constructed a multi-sequence learning network based on a Transformer, utilizing the multi-head self-attention mechanism to capture global features between long-distance segments. Similarly, Cao et al. [21] proposed an adaptive graph convolutional network that integrates temporal consistency and feature similarity of video segments to build global feature graphs. Wu et al. [22] further designed a dual-branch framework combining global and local modeling: the global branch captures long-distance dependencies using segment similarity priors, while the local branch excavates short-distance interactions within neighborhood frames.

### 2.3. Loss Function Constraint Strategy

Significant progress has been made in the design of loss functions under the MIL framework in recent years, with the core focus being on optimizing feature separability. Existing research primarily focuses on two strategies: The first strategy emphasizes enhancing class separability through bag-level constraints. For instance, Zhang et al. [23] proposed an intra-bag compactness loss to compress the feature distribution of normal instances, while Wan et al. [24] introduced a dynamic multi-instance loss combined with a center loss function, achieving feature space reconstruction via a Top-K selection strategy. The second strategy aims to strengthen discriminative power through boundary optimization. Yu et al. [25] designed a dynamic margin loss coupled with a verification loss mechanism to adaptively adjust decision boundaries, thereby improving model robustness. However, the above methods have not specifically targeted the mining and distinction of hard samples, failing to effectively address the issue of false alarms caused by hard samples. In the realm of loss function design, contrast learning has emerged as a potent approach in recent years. Aimed at learning discriminative feature representations, it operates by maximizing the similarity of positive sample pairs and minimizing that of negative ones in the feature space [26]. In the context of MIL, contrast learning can be integrated into loss function design to improve the model’s ability to distinguish between different instance categories. For example, the study [27] constructs positive and negative instance pairs within a bag, forcing the model to learn more discriminative features. Purwanto et al. [16] systematically expanded feature distances between positive and negative sample pairs by introducing a relative positioning loss, whereas Zhou et al. [28] proposed a joint metric loss employing an anchor-based strategy to dynamically adjust margins between positive and negative samples. However, similar to traditional MIL loss functions, most existing contrast learning methods in MIL struggle with hard sample pairs, where the feature differences between positive and negative samples are subtle. This leads to suboptimal performance in terms of false alarm reduction and accurate classification.

## 3. Proposed Method

**Figure 1:**
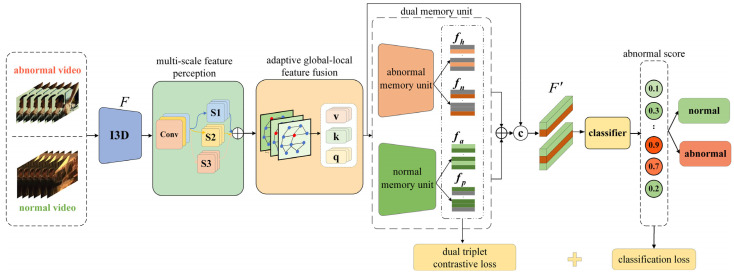
Framework of anomaly detection algorithm.

The proposed method consists of the multi-scale feature perception module, adaptive global–local feature fusion module, dual triplet contrastive loss module, and anomaly score prediction module, as illustrated in Figure 1. Initially, a set of normal and abnormal videos is processed through an I3D pretrained model to extract the initial feature F. Subsequently, the MFP module captures multi-scale features through convolutional layers with different receptive fields. Following this, the AGLF module adaptively fuses global and local patterns to capture both holistic structures and fine-grained details. Then, the dual-memory unit extracts four types of sample features $F^{\prime }$, which are used to compute the dual triplet contrastive loss function value, and concatenated to generate memory-augmented representations. Finally, the classifier generates anomaly scores for each video segment.

### 3.1. Multi-Scale Feature Perception

**Figure 2:**
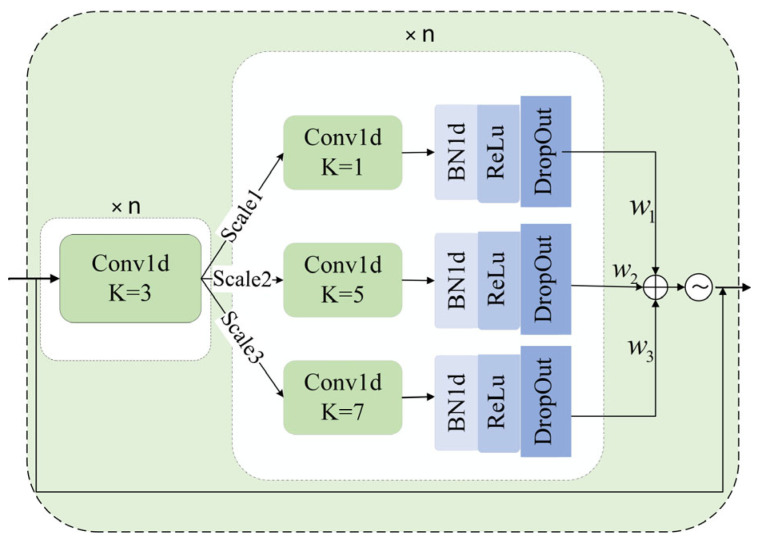
Network structure diagram of multi-scale feature perception module.

In the field of video anomaly detection, the diversity of object scales and the complexity of scenes pose key challenges. On the one hand, objects such as pedestrians and vehicles in videos exhibit significant scale differences, where subtle action details or grand scene changes may both serve as important indicators of abnormal events. On the other hand, complex scene factors, including occlusions and illumination variations, further exacerbate the difficulty of feature extraction. Traditional single-scale feature extraction patterns struggle to balance detail-level textures and global semantics simultaneously. When confronted with abrupt scale shifts in complex environments, such models tend to lose critical feature information, leading to substantial deterioration in detection accuracy. To overcome these limitations and comprehensively capture hierarchical semantic representations, we propose the multi-scale feature perception module. As depicted in Figure 2, the module employs convolutional kernels of different sizes on parallel branches to extract features with distinct receptive fields, which are then fused via learnable channel attention to capture more comprehensive multi-level feature information.

Initially, I3D features are enhanced through an n-layer 1D convolutional network, with each layer utilizing convolution operations with a kernel size of 3. Symmetric padding is applied to preserve the sequence length, and together with activation functions, progressively enhances the spatiotemporal expressive capability layer by layer. The specific structure is described as follows:

(1) $$H\left(x\right)=\sigma \left(T_{L}\left(\sigma \left(T_{L-1}\left(\ldots \sigma \left(T_{1}\left(x\right)\right)\ldots \right)\right)\right)\right)$$

(2) $$T_{i}\left(x\right)=\mathrm{Conv}\left(x,k=3,p=1\right)$$

Here, *T_i_*(*x*) denotes the convolutional operation at the *i*-th layer, *n* represents the number of convolutional layers, *x* corresponds to the I3D feature, *k* indicates the size of the convolutional kernel, *p* signifies the padding size, and σ refers to the ReLU activation function.

Subsequently, the features are fed into three parallel convolutional branches for processing: the 1 × 1 convolutional kernels focus on local fine-grained features, the 5 × 5 kernels extract mid-range contextual patterns, and the 7 × 7 kernels capture global semantic information. Each branch undergoes an iterative process of convolutional layers, batch normalization, activation functions, and Dropout layers for n times, forming a complementary multi-level feature representation. To further deepen feature fusion, a learnable channel attention mechanism is introduced, which performs a weighted sum of the feature between the three pathway branches, and a sigmoid activation function is used to generate an attention distribution map. Finally, this map is element-wise multiplied with the original I3D feature to suppress redundant information and amplify critical temporal-scale features. The specific structure is described as follows:

(3) $$P_{i}\left(x\right)=\sigma \left(\mathrm{BN}\left(\mathrm{Conv}\left(x,k=i,s=1,p=i//2\right)\right)\right)$$

(4) $$F_{out}=\sum_{m=1}^{3}\frac{\exp(\omega_{i})}{\sum_{i=1}^{3}\exp(\omega_{j})}\cdot P_{i}(x)+x$$

Here, *P_i_*(*x*) denotes the output of the *i*-th path, σ represents the Sigmoid activation function, $\omega_{i}$ corresponds to the weight of the *i*-th path, m indicates the path index, and *F*_out_ signifies the output of the module.

### 3.2. Adaptive Global–Local Feature Fusion

**Figure 3:**
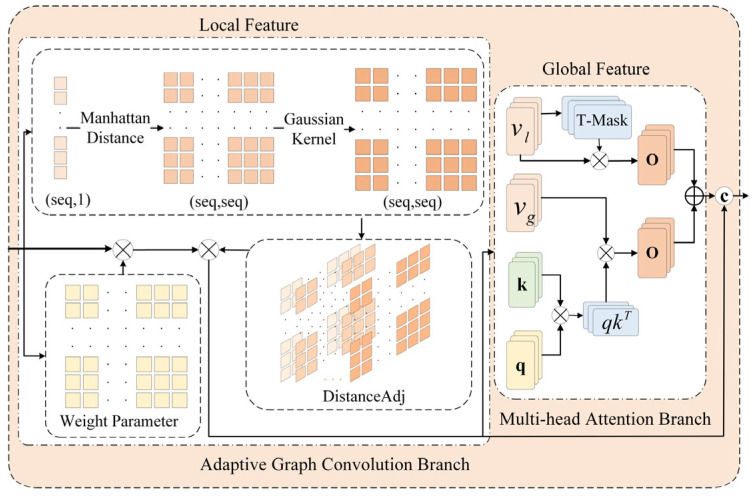
Network structure diagram of adaptive global–local feature fusion module.

Video data contains rich spatiotemporal information encompassing both long-range dependencies between instances and localized spatiotemporal correlations among adjacent frames, both of which are crucial for comprehensive video content understanding. Correspondingly, anomaly event detection relies on long-term spatiotemporal dynamics modeling to capture evolutionary trajectories and global patterns, while simultaneously requiring short-term fine-grained interactions to identify subtle anomalous features. However, traditional methods often fail to balance these aspects. Single attention mechanisms are good at capturing long-range dependencies but lack sensitivity to local, subtle changes. Single-graph convolutional networks can effectively extract local structural features but struggle to model complex long-range dependencies across instances. To overcome this limitation and achieve efficient complementary fusion of global dynamic patterns and local fine-grained features, thereby enhancing video content representation and comprehension, we construct an adaptive global–local feature fusion module. As shown in Figure 3, the module comprises an adaptive graph convolution branch (AGC) and a multi-head attention branch (MA), where the former mines spatiotemporal correlations within the local neighborhood of video frames, and the latter captures global dynamic features across long-distance segments. By implementing a gated mechanism-based feature fusion strategy, this module addresses the balancing challenge in modeling global–local feature relationships, achieving complementary feature enhancement.

In the adaptive graph convolution branch, the Manhattan distance matrix between elements is first computed based on the spatiotemporal sequence of input video segments. Then, the Gaussian kernel function is used to achieve a nonlinear smooth transformation of the distance values, and a weight decay mechanism is introduced to strengthen the correlation of long-distance positions, generating a spatially adaptive distance adjacency matrix (DistanceAdj). On this basis, tensor operations are performed between the learnable weight parameter matrix and the input feature, followed by matrix multiplication with the DistanceAdj to produce locally enhanced feature *F*_local_. The specific structure is described as follows:

(5) $$\mathrm{DistanceAdj}\left(\alpha ,N\right)=\exp\left(-\frac{\mathrm{Cityblock}\left(N\right)}{\exp\left(\alpha \right)}\right)$$

(6) $$F_{\mathrm{local}}\left(x\right)=\mathrm{Linear}\left(\sigma \left(\left(A\cdot xW\right)+b\right)+r\left(x\right)\right)$$

Here, α denotes a learnable parameter controlling the smoothing degree of the Gaussian kernel. *N* represents the sequence length that determines the shape of the adjacency matrix. Cityblock(*N*) indicates the Manhattan distance matrix computed among *N* nodes. *A* signifies the adjacency matrix, *W* corresponds to the learnable weight matrix, *b* refers to the bias term, σ represents the GELU activation function, and *r*(*x*) denotes the residual connection. *F*_local_ denotes the local features output by the graph convolutional branch.

The multi-head attention branch integrates relative positional encoding with the standard self-attention mechanism, where the former incorporates relative positional information between elements to account for content similarity in the sequence, while the latter captures long-distance dependencies and global patterns in video data. Initially, the input feature undergoes dimensional expansion through linear transformation, which quadruples the feature dimension before being partitioned into four components matching the original dimensionality, comprising a query vector, key vector, and two value vectors. Subsequently, the standard multi-head self-attention establishes global dependency relations through the dot product similarity calculation of query-key pairs, maintains numerical stability with a scaling factor, and generates attention weights for feature selection using the Softmax function. Simultaneously, the relative positional encoding attention constructs a learnable, exponentially decaying distance matrix to encode relative positional information into attention weights with spatial decay properties. Finally, the output feature from both pathways is concatenated, fused via a linear transformation, and output as globally enhanced features *F*_global_. The specific structure is described as follows:

(7) $$Atten_{1}\left(x\right)=\mathrm{Softmax}\left(\frac{QK^{T}}{\sqrt{d_{k}}}\right)V_{g}$$

(8) $$Atten_{2}\left(x\right)=\mathrm{Softmax}\left(\exp\left(-\frac{\left|i-j\right|}{\exp\left(1\right)}\right)\right)V_{l}$$

(9) $$F_{\mathrm{global}}\left(x\right)=\mathrm{Linear}\left(\mathrm{Cat}\left(Attn_{1}\left(x\right),Attn_{2}\left(x\right)\right)\right)$$

Here, *Attn*_1_(*x*) stands for standard multi-head attention; *Attn*_2_(*x*) indicates relative positional encoding attention; *Q*, *K*, and *V* represent the query vector, key vector, and value vector; d*_k_* specifies the dimensionality of the key vector; Cat refers to feature concatenation; and *F*_global_ denotes the global features output by the multi-head attention branch.

To achieve balanced representation between global and local features, this paper adopts a gated mechanism-based feature fusion strategy. Initially, the local and global features are concatenated at the feature dimension, and the spatially adaptive gated signal, gate, is generated through a gated network composed of linear layers and a Sigmoid activation function. Each element in the gate represents the relative importance of local and global features at the corresponding feature dimension. On this basis, gate and (1-gate) serve as adaptive weights to perform weighted fusion of local and global features, enabling dynamic adjustment of fusion weights according to the input feature distribution characteristics, thereby producing enhanced fused feature representations with complementary information.

(10) $$\mathrm{gate}=\mathrm{Sigmoid}\left(\mathrm{Linear}\left(\mathrm{Cat}\left(F_{local},F_{global}\right)\right)\right)$$

(11) $$F_{\mathrm{out}}=\mathrm{gate}\cdot F_{\mathrm{local}}+\left(1-\mathrm{gate}\right)\cdot F_{\mathrm{global}}$$

Here, gate denotes the gated signal, and *F*_out_ represents the output of the module.

### 3.3. Dual Triplet Contrastive Loss

**Figure 4:**
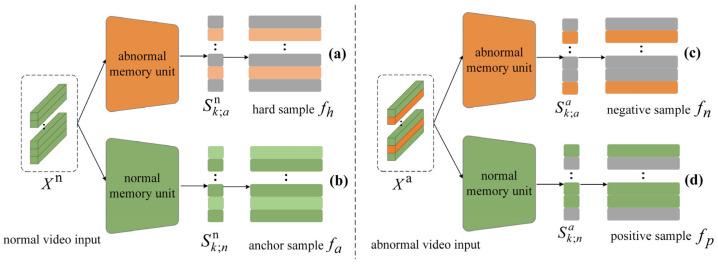
Feature extraction diagram of quadruple loss term. (**a**) The extraction process of hard samples. (**b**) The extraction process of anchor samples. (**c**) The extraction process of negative samples. (**d**) The extraction process of positive samples.

To address the challenges of hard sample mining and misclassification, this paper proposes a dual triplet contrastive loss strategy. By feeding normal and abnormal video features into a dual memory unit structure, four key features are extracted: hard sample, anchor sample, negative sample, and positive sample, with the extraction workflow illustrated in Figure 4. *X*^n^ represents the normal video features, and *X*^a^ represents the abnormal video features. The dual memory unit consists of a normal memory unit and an abnormal memory unit, both implemented as learnable two-dimensional parameter matrices, with dimensions corresponding to the number of memory units and the dimensionality of instance features, respectively. The normal memory unit stores learned normal behavioral patterns, while the abnormal memory unit retains learned abnormal behavioral patterns. S denotes a one-dimensional array that records the indices of the top-k instances with the highest abnormality scores, with its values mapping to the spatiotemporal positions in the video sequence that exhibit the most significant correlations after memory enhancement. Hard samples represent segments from normal videos that are prone to misclassification as abnormal behaviors. Anchor sample features correspond to the most representative normal behavioral features extracted from normal videos, which serve as anchor points in the loss function, attracting semantically similar normal instances while repelling abnormal ones. Negative sample features encapsulate the most discriminative abnormal features derived from abnormal videos. Positive sample features refer to normal behavioral features extracted from abnormal video data that exhibit strong correlations with the content of the normal memory unit, representing the substantial proportion of normal segments inherently present in anomaly-labeled videos.

As shown in Figure 4a, taking the extraction of hard sample features as an example.

Initially, the normal video features are multiplied by the storage matrix of the abnormal memory unit to generate an attention matrix with the number of instances as rows and the number of memory units as columns. This matrix reflects the association strength between each video instance and each memory unit. Subsequently, the Top-k operation is performed on each row of the attention matrix to select the attention scores of the k memory units most strongly correlated with that instance. These scores are averaged to produce a one-dimensional vector S with dimensionality equal to the number of instances, where each value represents the anomaly score of the corresponding instance enhanced by the memory units. Finally, another Top-k operation is applied to select the indices $S_{k;a}^{n}$ of the k instances with the highest anomaly scores. Based on these indices, the corresponding k instance features are extracted from the original normal video features, and their average is computed to obtain the feature representation of the hard sample.

(12) $$S=\mathrm{Mean}\left({\mathrm{Top}}_{k}\left(\mathrm{Attention}\left(x,M\right)\right),K\right)$$

(13) $$\mathrm{Attention}\left(x,M\right)=\mathrm{Softmax}\left(\frac{xM^{T}}{\sqrt{D}}\right)$$

(14) $$S_{k;a}^{n}=\arg{\mathrm{Top}}_{k}(S,K)$$

(15) $$F\left(S_{k;a}^{n},X^{n}\right)=\mathrm{Mean}\left(\mathrm{Gather}\left(X^{n},S_{k;a}^{n}\right)\right)$$

Here, *x* denotes the video feature; *M* represents the memory units; D indicates the feature dimension of the instance; Top*_k_*(.,*K*) denotes the selection of indices corresponding to the k highest attention values; and S represents a one-dimensional vector of anomaly scores for each instance. argTop*_k_*(.,*K*) denotes the operation of retrieving the indices of the k instances with the highest anomaly score, and $S_{k;a}^{n}$ represents the indices of the k instances with the highest anomaly score after normal video features are processed by abnormal memory units. Gather(*x*,.) signifies the extraction of elements based on the index values.

The extraction processes for the anchor sample, negative sample, and positive sample follow the workflow analogous to that of the hard sample. Figure 4b demonstrates the process of extracting anchor sample features by inputting normal video features into the normal memory unit; Figure 4c shows the process of extracting negative sample features by inputting abnormal video features into the abnormal memory unit; and Figure 4d illustrates the process of extracting positive sample features by inputting abnormal video features into the normal memory unit. The extraction process is represented as follows:

(16) $$f_{h}=F\left(S_{k;a}^{n};X^{n}\right)$$

(17) $$f_{a}=F\left(S_{k;n}^{n};X^{n}\right)$$

(18) $$f_{n}=F\left(S_{k;a}^{a};X^{a}\right)$$

(19) $$f_{p}=F\left(S_{k;n}^{a};X^{a}\right)$$

Here, X*^n^* denotes normal video features, and X*^a^* represents abnormal video features. In the notation S*_k_*;., the superscript denotes the input video type, and the subscript specifies the memory unit type. *f_h_*, *f_a_*, *f_n_*, and *f_p_* correspond to the feature representations of the hard sample, anchor sample, negative sample, and positive sample, respectively.

As shown in Equation (19), the proposed dual triplet contrastive loss constructs dual optimization objectives in the feature space through two combinations. The former ensures that normal features converge into a compact subspace, while the latter eliminates ambiguity in the boundary regions between hard samples and negative samples, forming a synergistic optimization effect during backpropagation. Notably, since the samples of each triplet are generated through both normal and abnormal memory units, the dual-triplet loss acts synchronously on the learnable parameter matrices of the dual memory units when performing optimization constraints, driving them to learn global-level normal and abnormal statistical features. The overall loss is defined as follows:

(20) $$L_{dual}=L\left(f_{a},f_{p},f_{n}\right)+L\left(f_{a},f_{h},f_{n}\right)$$

As illustrated in Equation (20), the first combination employs an anchor sample, negative sample, and positive sample to establish feature distance constraints. The designed contrastive loss computes the Euclidean distances between samples. If the sum of the distance between the positive sample and the anchor sample and the predefined margin exceeds the distance between the negative sample and the anchor sample, the loss function generates positive gradients. This mechanism automatically optimizes the parameter matrices of the memory units during backpropagation, driving positive samples to form compact distributions centered on the anchor points in the feature space while forcing negative samples to deviate beyond the margin m. This process enhances the intra-class aggregation of normal samples and also expands the separability between normal and abnormal features.

As shown in Equation (21), to address the ambiguity in boundary regions of the feature space, a second constraint is introduced by incorporating a hard sample. The contrastive loss function confines hard samples to the vicinity of anchor samples, establishing strong associations with normal features, while simultaneously pushing negative samples away from this region. This mechanism effectively eliminates distribution overlaps between hard samples and abnormal features in boundary regions by separating ambiguous hard samples from abnormal patterns in the feature space.

(21) $$L\left(f_{a},f_{p},f_{n}\right)=\ln\left(1+\exp\left(\sqrt{\sum_{i=1}^{n}{\left(a_{i}-p_{i}\right)}^{2}}-\sqrt{\sum_{i=1}^{n}{\left(a_{i}-n_{i}\right)}^{2}}+m\right)\right)$$

(22) $$L\left(f_{a},f_{h},f_{n}\right)=\ln\left(1+\exp\left(\sqrt{\sum_{i=1}^{n}{\left(a_{i}-h_{i}\right)}^{2}}-\sqrt{\sum_{i=1}^{n}{\left(a_{i}-n_{i}\right)}^{2}}+m\right)\right)$$

Here, *a* denotes the anchor sample, *p* represents the positive sample, *n* corresponds to the negative sample, *h* signifies the hard sample, and *m* indicates the predefined margin.

### 3.4. Overall Loss Functions

Under the weakly supervised task setting at the video level, our classification loss is implemented using binary cross-entropy calculated with video-level labels. For each video *V* in the test set, the model predicts anomaly scores $S=s_{1},\ldots s_{t}$ for each instance segment. The video-level predicted label $\overset{\wedge }{y}$ is defined as the average of the top k anomaly scores. The ground-truth label is denoted as *y*, where 0 indicates normal and 1 represents anomaly. The classification loss is defined as:

(23) $$L_{cls}=-\frac{1}{B}\sum_{i=1}^{B}\left(y_{i}\log(\overset{\wedge }{y_{i}})+(1-y_{i})\log(1-{\overset{\wedge }{y}}_{i})\right)$$

where B represents the batch size. Overall, combining the dual triplet contrastive loss, the loss function for our proposed method is as follows:

(24) $$L=\lambda_{1}L_{cls}+\lambda_{2}L_{dual}$$

## 4. Experiments

### 4.1. Datasets and Evaluation Metrics

Datasets. Similar to previous works, the proposed method has been experimentally evaluated on two large-scale video anomaly detection datasets: UCF-Crime [3] and XD-Violence [22].

XD-Violence is a multi-scenario video dataset specifically designed for multimodal video anomaly detection. This comprehensive dataset aggregates video samples from diverse real-world contexts, including surveillance footage, cinematic content, and sports broadcasts, covering six categories of anomalous events: abuse, explosions, car accidents, fighting, shooting, and riots. With a total duration of 217 h, the dataset contains 4754 untrimmed video clips equipped with audio signals. Both training and test sets include normal and anomalous videos, with video-level annotations provided for the training set and frame-level labels for the test set.

UCF-Crime consists of 1900 continuous, untrimmed surveillance videos sourced from diverse scenarios, time periods, and lighting conditions, encompassing 13 common anomalous events, including abuse, burglary, robbery, and related incidents. Both training and test sets contain normal and anomalous videos, with video-level annotations provided for 1610 training samples and frame-level labels applied to 290 test samples. The complete collection spans 128 h of footage, serving as a large-scale benchmark for weakly supervised video anomaly detection tasks.

Evaluation Metrics. Experiments utilize the area under the curve (AUC) of the frame-level receiver operating characteristic (ROC) and the average precision (AP) as evaluation metrics for the UCF-Crime and XD-Violence datasets. These metrics are computed using the frame-level predicted anomaly scores from the model outputs and the corresponding frame-level ground truth labels. The False Alarm Rate (FAR) quantifies the likelihood of the model misclassifying normal videos as anomalous, computed using all normal video samples in the test set. The AP*_sub_* and AUC*_sub_* specifically evaluate the anomalous video detection performance of the model, with calculations exclusively based on all abnormal video samples in the test set. Additionally, computational overhead, computational efficiency, and parameter count are measured to comprehensively assess the practical performance of the module.

### 4.2. Implementation Details

The video streams from the XD-Violence and UCF-Crime are decoded into frame sequences, resized to a spatial resolution of 224 × 224, and normalized to pixel values within the range [−1, 1]. To enhance spatial diversity, 5-crop and 10-crop augmentation strategies were applied, respectively. The frames are partitioned into non-overlapping segments of 16 frames using a fixed time step, and each segment is input into the I3D network model pretrained on Kinetics-400 to extract a 1024-dimensional feature vector.

During the training phase, the data loader is configured to limit the number of video segments M to 200. The model is trained in an end-to-end manner using the Adam optimizer with beta coefficients of (0.9, 0.999), a weight decay coefficient of 0.00005, and a learning rate of 0.0001. The value of k for the Top-k operation is 4. The batch size is set to 64, and the number of iterations is 3000.

### 4.3. Comparison Study

#### 4.3.1. Comparison with SOTA Methods

Under identical experimental settings with default parameters and pretrained features, a comparative results analysis is conducted with other methods on two widely used benchmark datasets.

Table 1 presents the AP scores of the proposed method and other methods on the XD-Violence. The AP score of the proposed method reaches 83.47%, outperforming other model approaches. In comparison with methods that use the same I3D pretrained network to extract RGB features, namely RTFM [15], CMRL [29], DMU [28], and Tan et al. [30], the proposed method achieves performance improvements of 5.66%, 2.17%, 3.62%, and 1.37%, respectively. The S3R [31] and CUPL [32] methods employ two-stage self-learning strategies with generated pseudo-labels, and the proposed method shows significant enhancements over both. Furthermore, even when compared to MSL [20] and MGFN [8], which employ larger-scale VideoSwin pretrained networks for RGB feature extraction, the proposed method achieves enhanced performance with improvements of 4.88% and 3.36%, respectively.

Table 2 presents a comparison of AUC scores for the proposed method against other methods on the UCF-Crime. The AUC score of the proposed method reaches 87.16%, outperforming other model approaches. In comparison with methods using the same I3D pretrained network for RGB feature extraction, namely RTFM [15], S3R [31], DMU [28], and CMRL [29], the proposed method achieves performance improvements of 2.86%, 1.17%, 1.15%, and 1.06%, respectively. Compared to OPVAD [34], which employs the CLIP multi-modal contrastive pretrained model for RGB feature extraction, the proposed method yields an improvement of 1.11%. Compared to the two-stage pseudo-label generation strategy of CUPL [32], the proposed method achieves a 0.94% performance gain. Even when against MSL [20], which leverages VideoSwin pretrained networks for enhanced feature extraction, the proposed method maintains a competitive edge with a 1.54% improvement.

#### 4.3.2. Comparative Study of Feature Extraction Methods

To validate the effectiveness of the proposed AGLF module in discriminative feature extraction, this study conducts comparative experiments with two classical multi-scale feature extraction methods: Pyramidal Temporal Frame Prediction (PTFP) [37] and Dilated Multi-scale Convolution (DMC) [38]. The PTFP method constructs the multi-granularity pyramidal spatiotemporal architecture, employing dynamic adaptive scale selection and cross-layer gated feature interaction for fusion. The DMC method designs the multi-level dilated convolution module combined with adaptive receptive field selection to capture multi-scale spatiotemporal features. As shown in Table 3, the proposed method demonstrates significant advantages across multiple key metrics. Compared to the DMC method, the AUC scores on UCF-Crime are improved by 1.6%, and the AP scores on XD-Violence are increased by 0.63%. Simultaneously, the model parameters are reduced by approximately 10.3%, and computational overhead is decreased by 10.2%, fully validating the effectiveness of the AGLF module in improving computational efficiency and feature representation capability.

### 4.4. Ablation Study

#### 4.4.1. Effectiveness of Different Components

To comprehensively evaluate the effectiveness of individual components in the proposed method, an ablation study is conducted by progressively integrating all possible combinations of components, and the results are presented in Table 4. In the dual triplet contrastive loss strategy, the dual memory units employed for key sample selection are denoted as M*_dual_*. To further validate the critical role of M*_dual_*, we conduct comparative experiments using a single-layer binary classifier, denoted as Cl*_sin_*, to replace the dual memory units for key sample selection.

When only the MFP module is added, the most significant improvement in detection accuracy is observed. The AUC and AUC*_sub_* scores for UCF-Crime increase by 0.41% and 0.04%, respectively, while the AP and AP*_sub_* scores for XD-Violence improve by 2.07% and 1.51%. This demonstrates the critical role of multi-scale feature extraction in distinguishing between normal and abnormal instances. When only the AGLF module is incorporated, the AUC score for UCF-Crime increases by 0.12%, and the AP and AP*_sub_* scores for XD-Violence increase by 1.41% and 1.06%, respectively, proving that the local–global feature fusion enhances the discriminative feature representation for anomaly and normality. Compared with only using the Cl*_sin_*, employing only the M*_dual_* significantly lowers the false alarm rate of the model. The FAR score for UCF-Crime decreases by 1.33%, and the AP and AP*_sub_* scores for XD-Violence increase by 2.49% and 2.23%, respectively, with a decrease in the FAR score by 1.49%. This confirms that the storage properties of dual memory units enable superior selection of key samples, thereby enhancing the model’s capacity to learn discriminative features.

When the MFP module is combined with the AGLF module, the AUC score for UCF-Crime reaches 86.11%. When the MFP module is combined with the M*_dual_*, the AP and APD scores for XD-Violence reach 81.92% and 82.97%, respectively, with a significant reduction in the FAR score to 0.41%. Similarly, combining AGLF with the M*_dual_* strategy reduces the FAR score on XD-Violence to 0.41%. These results demonstrate that different component combinations mutually complement each other, collectively enhancing model performance.

When the three components are integrated, the combination containing M*_dual_* outperforms the combination containing Cl*_sin_*, and the comprehensive performance of the model reaches the optimal level. The AUC and AUC*_sub_* scores for UCF-Crime increase by 1.38% and 1.92%, respectively, with a FAR reduction of 0.65%. For XD-Violence, the AP and AP*_sub_* scores improve by 3.62% and 2.9%, respectively, while the FAR decreases by 0.77%. This synergy confirms that the proposed components collaboratively leverage their complementary advantages to enhance anomaly detection capabilities, ultimately validating the effectiveness of our method.

#### 4.4.2. Effectiveness of Different Loss

To validate the synergistic constraint effectiveness of the proposed dual triplet contrastive loss strategy, an ablation study on the loss function terms is conducted, as shown in Table 5. Here, *L*_cls_ denotes the base classification loss computed by the cross-entropy function between predicted and ground-truth labels. When individually adding either loss term, the model exhibits consistent improvements in anomaly detection performance. Notably, the *L_(a,h,n)_* term demonstrates more pronounced reductions in FAR, confirming its efficacy in constraining feature representations of hard samples. When both loss terms are added simultaneously, the AUC score for UCF-Crime increases by 4.45%, and the FAR score decreases by 0.96%. For XD-Violence, the AP score increases by 1.68%, and the FAR score decreases by 0.45%. The experimental results demonstrate the synergistic constraint effects of the dual triplet contrastive loss strategy and validate its efficacy in addressing false alarms caused by hard samples.

#### 4.4.3. Different Fusion Strategies

To verify the effectiveness of the proposed gated mechanism-based feature fusion strategy in enhancing the complementarity between local and global features, ablation experiments are conducted comparing different feature fusion approaches. As shown in Table 6, the gated fusion strategy achieves AUC and AP scores of 87.16% and 83.47% on the UCF-Crime and XD-Violence datasets, respectively, outperforming cross-attention, element-wise multiplication, and additive fusion strategies while requiring only half the parameters of the cross-attention approach. Additionally, the gated fusion strategy demonstrated superior performance compared to individual feature branches alone, validating that the proposed strategy successfully integrates the distinct advantages of local and global features to achieve complementary feature enhancement.

### 4.5. Hyperparameter Sensitivity Analysis

**Figure 5:**
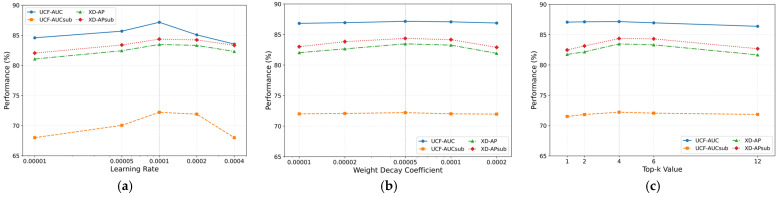
Sensitivity analysis of model hyperparameters. (**a**) Impact of learning rate on model performance. (**b**) Impact of weight decay coefficient on model performance. (**c**) Impact of Top-k value on model performance.

This section of the experiment analyzes the impact of key hyperparameters, including learning rate, weight decay coefficient, and Top-k value, on model performance. As shown in Figure 5a, the learning rate significantly influences model performance, exhibiting an initial increase followed by a decrease as the learning rate grows. When the learning rate reaches 0.0004, the training process diverges and converges to local optima, resulting in substantial performance degradation. Figure 5b demonstrates that the weight decay coefficient, serving as a crucial regularization technique to prevent overfitting, also impacts model performance. Optimal overall performance is achieved at 0.00005, with deviations causing marginal declines across evaluation metrics. As shown in Figure 5c, the selection of Top-k value is also crucial. A smaller k value limits the number of key samples selected, causing the model to be unable to fully learn the feature. On the other hand, a larger k value introduces redundant samples, which interfere with the model’s learning of discriminative features. The experimental results show that the optimized configuration of learning rate, weight decay coefficient, and Top-k value has an important impact on model performance, and the optimal values are determined as 0.0001, 0.00005, and 4, respectively, which effectively ensures that the model achieves a robust and excellent performance.

### 4.6. Training Trajectory Analysis on Feature Distance Evolution

**Figure 6:**
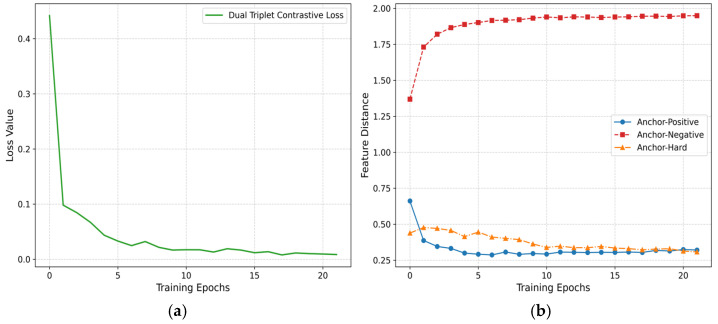
Training dynamics of dual triplet contrastive loss on XD-Violence. (**a**) Loss convergence. (**b**) Feature distance trajectories.

**Figure 7:**
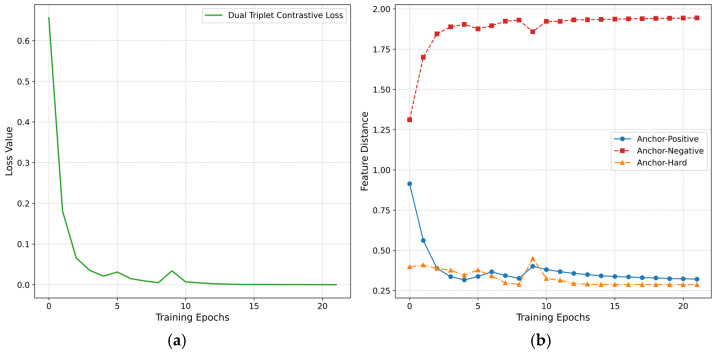
Training dynamics of dual triplet contrastive loss on UCF-Crime. (**a**) Loss convergence. (**b**) Feature distance trajectories.

To intuitively elucidate the mechanistic role of the dual triplet contrastive loss function during model training, this study conducts a visual analysis of the loss values and the evolving feature distances between sample pairs on the XD-Violence and UCF-Crime datasets. As shown in Figure 6 and Figure 7, the contrastive loss exhibits a declining trend as training epochs progress, eventually converging after the 20th epoch, which validates the stability of the optimization process. The feature distances between the anchor and positive sample, as well as between the anchor and hard sample, gradually decrease and stabilize, while the distance between the anchor and negative sample progressively increases until reaching equilibrium. The differentiated evolution of the three types of sample distances results in an increasingly enlarged degree of separation, illustrating the evolutionary process of the mechanism behind the loss function strategy.

### 4.7. Qualitative Results

#### 4.7.1. Feature Distribution Analysis via t-SNE

**Figure 8:**
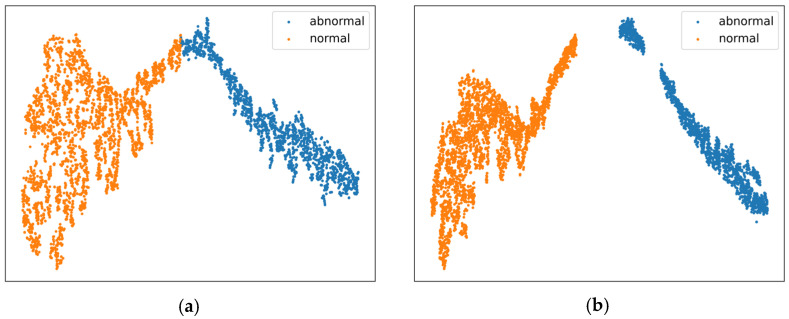
Visualization of feature distribution on XD-Violence. (**a**) Baseline. (**b**) Ours.

**Figure 9:**
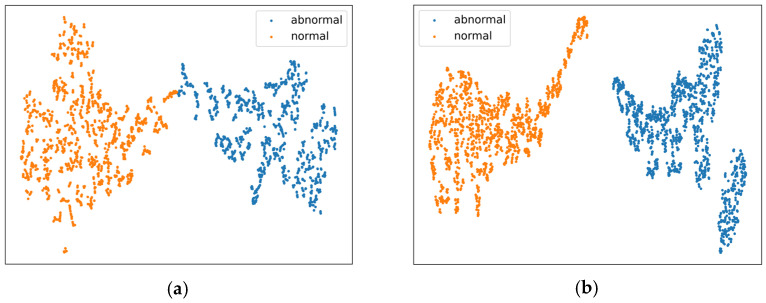
Visualization of feature distribution on UCF-Crime. (**a**) Baseline. (**b**) Ours.

To verify the effectiveness of the modules and loss strategies proposed in enhancing the discriminative features between normal and abnormal events, an experiment was conducted using the t-SNE method [39]. The classification features extracted by the baseline and improved models are compared and analyzed on the XD-Violence and UCF-Crime test sets, with the results presented in Figure 8 and Figure 9. The experiment initially retrieves the indices of the maximum segment-level anomaly scores output by the classifier, then extracts the classification features corresponding to these segments, which are considered the most representative normal or abnormal features in the videos. These features then undergo dimensionality reduction and visualization. In the figures, orange dots represent normal features, and blue dots represent abnormal features. Through a comparative analysis of the left and right subfigures, the proposed method demonstrates significant improvements in effectively distinguishing between normal and abnormal discriminative features.

#### 4.7.2. Distribution of Pairwise Sample Distances

**Figure 10:**
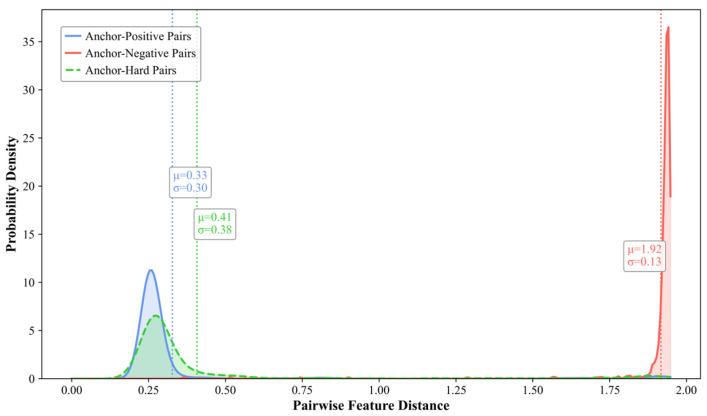
Probability density distribution of feature distances between sample pairs on XD-Violence.

**Figure 11:**
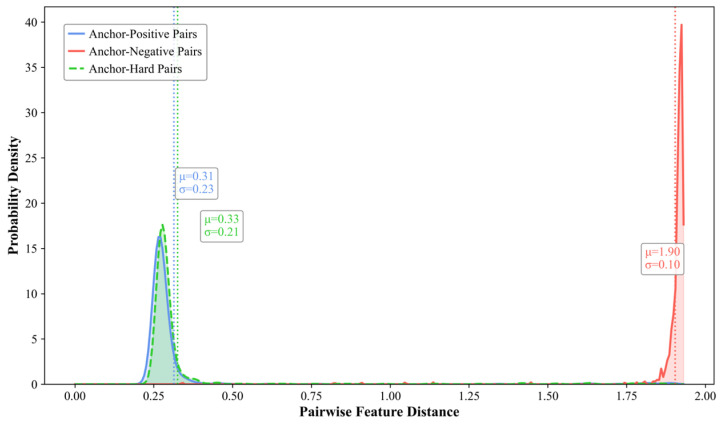
Probability density distribution of feature distances between sample pairs on UCF-Crime.

To intuitively demonstrate the constraint mechanism of the dual triplet contrastive loss function, the probability density distributions of feature distances between sample pairs are visualized on the XD-Violence and UCF-Crime test sets, as shown in Figure 10 and Figure 11. Taking the results of the XD-Violence test set as an example, by calculating the mean (μ) and standard deviation (σ) of the feature distance between anchor samples and the three types of sample pairs, the results reveal that the mean feature distance between anchor samples and negative sample pairs (μ = 1.92) is significantly greater than those of positive sample pairs (μ = 0.33) and hard sample pairs (μ = 0.41), indicating that abnormal features are constrained within narrow regions far from the anchor points. Simultaneously, the proximity of mean distances between hard sample pairs and positive sample pairs suggests that hard samples are confined within the normal feature space, clustering near the normal feature centroids.

#### 4.7.3. Anomaly Scores

**Figure 12:**
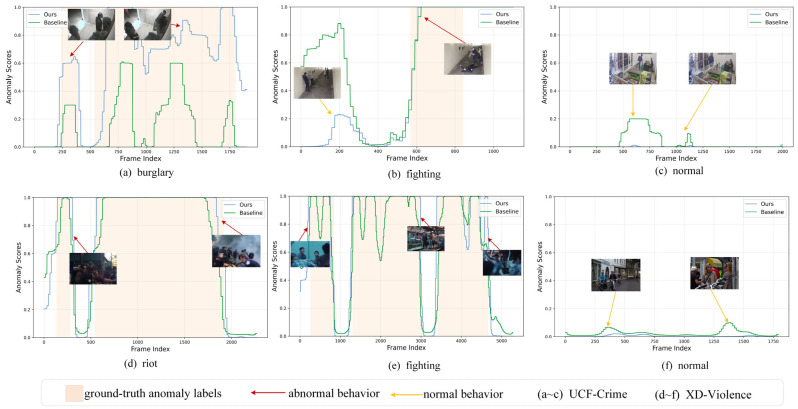
Frame-level anomaly score visualization.

To intuitively demonstrate the effectiveness of the proposed method in improving anomaly detection accuracy and reducing false alarm rate, experiments are conducted on video samples from the UCF-Crime and XD-Violence test sets using the anomaly score visualization method [40]. The results are illustrated in Figure 12. In the figure, the horizontal axis represents the video frame index, and the vertical axis indicates the anomaly score for the corresponding frame, with the range being [0, 1]. Orange-shaded regions indicate ground-truth anomalous frame intervals. Green and blue curves depict anomaly scores from the baseline model and the improved model, respectively. Red and yellow arrows mark the temporal positions of anomalous and normal behavior scenes.

In Figure 12a,d, the blue curve yields higher anomaly prediction scores within the orange-shaded regions, and all anomaly scores exceed the threshold, more closely adhering to the orange background area. This indicates that the improved model is more effective in detecting anomalies. Figure 12b,e indicate that at the boundary of the orange-shaded regions, the blue curve shows more abrupt transitions, effectively distinguishing the boundary between anomalous and normal behavior. Concurrently, the anomaly prediction scores are lower in the normal region, accurately identifying anomalies and avoiding false alarms in normal areas. Figure 12c,f display the predictions for normal videos, where the blue curve has lower anomaly prediction scores, indicating an effective reduction in the anomaly prediction scores for hard samples and a decrease in the probability of model false alarms.

## 5. Conclusions

This paper proposes a video anomaly detection method based on dual triplet contrastive loss constraints for hard instances, effectively addressing the challenges of hard sample mining and misclassification. The method employs dual memory units to extract the features of negative samples, anchor samples, positive samples, and hard samples, and utilizes a contrastive loss function to constrain feature space distributions, significantly enhancing the model’s discriminative capability between hard samples and abnormal features. By integrating the hierarchical feature extraction capacity of the multi-scale feature perception module with the spatiotemporal modeling advantages of the adaptive global–local feature fusion module, a highly discriminative feature representation system is constructed. Experimental results demonstrate that the proposed method maintains high detection accuracy while effectively reducing false alarm rates. In future research, we will further investigate the complex interactions between hard samples and the other three sample types through contrastive learning approaches.

## Figures and Tables

**Table 1:** Comparison of experimental results on the XD-Violence dataset.

Method	Feature	AP (%)
RTFM [15]	I3D-RGB	77.81
MSL [20]	VideoSwin-RGB	78.59
S3R [31]	I3D-RGB	80.26
MGFN [8]	VideoSwin-RGB	80.11
CMRL [29]	I3D-RGB	81.30
CUPL [32]	I3D-RGB	81.43
DMU [28]	I3D-RGB	79.85
REWARD-E2E [33]	Uniformer-RGB	80.30
Tan et al. [30]	I3D-RGB	82.10
Ours	I3D-RGB	83.47

**Table 2:** Comparison of experimental results on the UCF-Crime dataset.

Method	Feature	AUC (%)
RTFM [15]	I3D-RGB	84.30
CTR [35]	I3D-RGB	84.89
MSL [20]	VideoSwin-RGB	85.62
S3R [31]	I3D-RGB	85.99
DMU [28]	I3D-RGB	86.01
Park et al. [36]	I3D-RGB	85.63
OPVAD [34]	CLIP-RGB	86.05
CMRL [29]	I3D-RGB	86.10
CUPL [32]	I3D-RGB	86.22
Ours	I3D-RGB	87.16

**Table 3:** Performance comparison results of feature extraction methods.

Module	UCF (%)					XD (%)					GFLOPs	TFLOPs/s	Params (M)
	AUC	AP	AUC*_sub_*	AP*_sub_*	FAR	AUC	AP	AUC*_sub_*	AP*_sub_*	FAR			
PTFP	84.14	21.07	62.61	23.60	1.63	93.34	80.18	80.59	81.34	0.93	2.14	3.47	10.90
DMC	85.56	37.07	68.82	39.46	0.99	94.27	82.84	83.12	83.69	0.56	2.05	3.22	10.23
Our	87.16	34.14	72.21	36.61	0.71	94.40	83.47	83.81	84.36	0.45	1.84	3.92	9.18

**Table 4:** Ablation study of proposed components.

Module				Dataset									
				UCF (%)					XD (%)				
MFP	AGLF	Cl*_sin_*	M*_dual_*	AUC	AP	AUC*_sub_*	AP*_sub_*	FAR	AUC	AP	AUC*_sub_*	AP*_sub_*	FAR
				85.78	31.85	70.29	34.59	1.36	93.10	79.85	81.02	81.46	1.22
√				86.19	32.94	70.33	35.36	1.34	93.88	81.92	82.22	82.97	0.41
	√			85.90	32.81	69.92	35.29	1.26	93.71	81.26	82.28	82.52	0.94
		√		83.02	29.64	67.09	33.43	2.03	91.32	77.97	80.13	79.62	1.89
			√	85.50	31.91	69.51	34.69	0.70	93.29	80.46	81.32	81.85	0.40
	√		√	85.63	32.94	70.72	35.88	1.04	93.77	81.35	81.84	82.31	0.49
√	√			86.11	32.54	70.07	34.93	1.21	93.65	80.61	81.51	81.68	0.97
√			√	85.81	33.14	70.34	35.85	0.75	90.13	81.92	82.22	82.97	0.41
√	√	√		84.86	31.35	69.93	34.72	1.91	91.29	80.73	80.52	81.64	1.76
√	√		√	87.16	34.14	72.21	36.61	0.71	94.40	83.47	83.81	84.36	0.45

**Table 5:** Ablation studies on different loss terms.

Module				Dataset									
				UCF (%)					XD (%)				
L*_cls_*	L*_(a,p,n)_*	L*_(a,h,n)_*	L*_dua_*_l_	AUC	AP	AUC*_sub_*	AP*_sub_*	FAR	AUC	AP	AUC*_sub_*	AP*_sub_*	FAR
√				82.71	26.03	59.40	27.30	1.67	94.12	81.79	81.65	82.19	0.90
√	√			86.11	32.54	70.07	34.93	0.89	93.90	82.07	81.26	81.73	0.56
√		√		83.06	29.24	61.18	30.59	0.83	94.01	82.40	80.96	81.33	0.52
√			√	87.16	34.14	72.21	36.61	0.71	94.40	83.47	83.81	84.36	0.45

**Table 6:** Ablation studies on different fusion strategies.

Fusion Strategy	UCF (%)					XD (%)					Params (k)
	AUC	AP	AUC*_sub_*	AP*_sub_*	FAR	AUC	AP	AUC*_sub_*	AP*_sub_*	FAR	
AGC branch	84.21	31.76	65.84	33.45	2.08	92.91	79.95	82.04	81.72	1.96	_
MA branch	85.99	34.27	70.99	36.93	2.63	93.84	81.28	81.95	82.18	1.79	_
CrossAttention	81.17	24.14	58.26	25.71	2.40	91.50	72.19	73.27	72.63	1.83	1048.6
ElementwiseMultiply	85.58	34.70	71.18	37.52	1.21	92.83	81.36	83.21	83.75	1.67	0
Addition	86.35	35.53	71.53	38.36	0.95	94.01	82.38	82.47	83.45	0.53	0
GatedFusion	87.16	34.14	72.21	36.61	0.71	94.40	83.47	83.81	84.36	0.45	524.8

## Data Availability

The original contributions presented in this study are included in the article. Further inquiries can be directed to the corresponding author. The code is available at https://github.com/ncxa/DTCL (accessed on 24 April 2025).

## References

[1] Li W., Mahadevan V., Vasconcelos N. (2013). Anomaly detection and localization in crowded scenes. IEEE Trans. Pattern Anal. Mach. Intell..

[2] Wu P., Pan C., Yan Y., Pang G., Wang P., Zhang Y. (2024). Deep learning for video anomaly detection: A review. arXiv.

[3] Sultani W., Chen C., Shah M. Real-world anomaly detection in surveillance videos. Proceedings of the IEEE Conference on Computer Vision and Pattern Recognition.

[4] Zhou Y., Qu Y., Xu X., Shen F., Song J., Shen H. (2023). BatchNorm-based Weakly Supervised Video Anomaly Detection. arXiv.

[5] Pu Y., Wu X., Yang L., Wang S. (2024). Learning prompt-enhanced context features for weakly-supervised video anomaly detection. IEEE Trans. Image Process..

[6] Dang Y., Chen J., Chen P., Gao N., Huan R., Zhao D. (2024). Generate anomalies from normal: A partial pseudo-anomaly augmented approach for video anomaly detection. Vis. Comput..

[7] Zhang L., Li S., Luo X., Liu X., Zhang R. (2024). Video anomaly detection with both normal and anomaly memory modules. Vis. Comput..

[8] Chen Y., Liu Z., Zhang B., Fok W., Qi X., Wu Y.C. Mgfn: Magnitude-contrastive glance-and-focus network for weakly-supervised video anomaly detection. Proceedings of the AAAI Conference on Artificial Intelligence.

[9] Robinson J., Chuang C.Y., Sra S., Jegelka S. (2020). Contrastive learning with hard negative samples. arXiv.

[10] Zhang H., Li Z., Yang J., Wang X., Guo C., Feng C. (2023). Revisiting Hard Negative Mining in Contrastive Learning for Visual Understanding. Electronics.

[11] Zhao Y., Shu Q. (2025). Debiased hybrid contrastive learning with hard negative mining for unsupervised person re-identification. Digit. Signal Process..

[12] Gong Y., Wang C., Dai X., Yu S., Xiang L., Wu J. (2022). Multi-scale continuity-aware refinement network for weakly supervised video anomaly detection. Proceedings of the 2022 IEEE International Conference on Multimedia and Expo (ICME).

[13] Ristea N.C., Madan N., Ionescu R.T., Nasrollahi K., Khan F.S., Moeslund T.B., Shah M. Self-supervised predictive convolutional attentive block for anomaly detection. Proceedings of the IEEE/CVF Conference on Computer Vision and Pattern Recognition.

[14] Gong S., Chen Y. (2022). Video action recognition based on spatio-temporal feature pyramid module. Proceedings of the 2020 13th International Symposium on Computational Intelligence and Design (ISCID).

[15] Tian Y., Pang G., Chen Y., Singh R., Verjans J.W., Carneiro G. Weakly-supervised video anomaly detection with robust temporal feature magnitude learning. Proceedings of the IEEE/CVF International Conference on Computer Vision.

[16] Purwanto D., Chen Y.T., Fang W.H. Dance with self-attention: A new look of conditional random fields on anomaly detection in videos. Proceedings of the IEEE/CVF International Conference on Computer Vision.

[17] Rahimpour S.M., Kazemi M., Moallem P., Safayani M. (2024). Video anomaly detection based on attention and efficient spatio-temporal feature extraction. Vis. Comput..

[18] Pu Y., Wu X. (2022). Locality-aware attention network with discriminative dynamics learning for weakly supervised anomaly detection. Proceedings of the 2022 IEEE International Conference on Multimedia and Expo ICME.

[19] Naji Y., Setkov A., Loesch A., Gouiffès M., Audigier R. (2022). Spatio-temporal predictive tasks for abnormal event detection in videos. Proceedings of the 2022 18th IEEE International Conference on Advanced Video and Signal Based Surveillance (AVSS).

[20] Li S., Liu F., Jiao L. Self-training multi-sequence learning with transformer for weakly supervised video anomaly detection. Proceedings of the AAAI Conference on Artificial Intelligence.

[21] Cao C., Zhang X., Zhang S., Wang P., Zhang Y. (2022). Adaptive graph convolutional networks for weakly supervised anomaly detection in videos. IEEE Signal Process. Lett..

[22] Wu P., Liu J., Shi Y., Sun Y., Shao F., Wu Z., Yang Z. (2020). Not only look, but also listen: Learning multimodal violence detection under weak supervision. Proceedings of the Computer Vision–ECCV 2020: 16th European Conference.

[23] Zhang J., Qing L., Miao J. (2019). Temporal convolutional network with complementary inner bag loss for weakly supervised anomaly detection. Proceedings of the 2019 IEEE International Conference on Image Processing (ICIP).

[24] Wan B., Fang Y., Xia X., Mei J. (2020). Weakly supervised video anomaly detection via center-guided discriminative learning. Proceedings of the 2020 IEEE International Conference on Multimedia and Expo (ICME).

[25] Yu S., Wang C., Mao Q., Li Y., Wu J. (2021). Cross-epoch learning for weakly supervised anomaly detection in surveillance videos. IEEE Signal Process. Lett..

[26] Chen T., Kornblith S., Norouzi M., Hinton G. A simple framework for contrastive learning of visual representations. Proceedings of the International Conference on Machine Learning.

[27] Doersch C., Gupta A., Efros A.A. Unsupervised visual representation learning by context prediction. Proceedings of the IEEE International Conference on Computer Vision.

[28] Zhou H., Yu J., Yang W. Dual memory units with uncertainty regulation for weakly supervised video anomaly detection. Proceedings of the AAAI Conference on Artificial Intelligence.

[29] Cho M., Kim M., Hwang S., Park C., Lee K., Lee S. Look around for anomalies: Weakly-supervised anomaly detection via context-motion relational learning. Proceedings of the IEEE/CVF Conference on Computer Vision and Pattern Recognition.

[30] Tan W., Yao Q., Liu J. Overlooked video classification in weakly supervised video anomaly detection. Proceedings of the IEEE/CVF Winter Conference on Applications of Computer Vision.

[31] Wu J.C., Hsieh H.Y., Chen D.J., Fuh C.S., Liu T.L. (2022). Self-supervised sparse representation for video anomaly detection. Proceedings of the European Conference on Computer Vision.

[32] Zhang C., Li G., Qi Y., Wang S., Qing L., Huang Q., Yang M.H. Exploiting completeness and uncertainty of pseudo labels for weakly supervised video anomaly detection. Proceedings of the IEEE/CVF Conference on Computer Vision and Pattern Recognition.

[33] Karim H., Doshi K., Yilmaz Y. Real-time weakly supervised video anomaly detection. Proceedings of the IEEE/CVF Winter Conference on Applications of Computer Vision.

[34] Wu P., Zhou X., Pang G., Sun Y., Liu J., Wang P., Zhang Y. Open-vocabulary video anomaly detection. Proceedings of the IEEE/CVF Conference on Computer Vision and Pattern Recognition.

[35] Wu P., Liu J. (2021). Learning causal temporal relation and feature discrimination for anomaly detection. IEEE Trans. Image Process..

[36] Park S., Kim H., Kim M., Kim D., Sohn K. Normality guided multiple instance learning for weakly supervised video anomaly detection. Proceedings of the IEEE/CVF Winter Conference on Applications of Computer Vision.

[37] Javed M.H., Li T., Yu Z., Hussain A., Rajeh T.M., Zhang F. (2023). Pyramidal temporal frame prediction for efficient anomalous event detection in smart surveillance systems. Knowl.-Based Syst..

[38] Tahira N.J., Park J.R., Lim S.J., Park J.S. (2023). YOLOv5 based Anomaly Detection for Subway Safety Management Using Dilated Convolution. J. Korean Soc. Ind. Converg..

[39] Van der Maaten L., Hinton G. (2008). Visualizing data using t-SNE. J. Mach. Learn. Res..

[40] Chen W., Ma K.T., Yew Z.J., Hur M., Khoo D.A.A. TEVAD: Improved video anomaly detection with captions. Proceedings of the IEEE/CVF Conference on Computer Vision and Pattern Recognition.

